# The Food Warden: An Exploration of Issues in Distributing Responsibilities for Safe-by-Design Synthetic Biology Applications

**DOI:** 10.1007/s11948-017-9969-0

**Published:** 2017-09-26

**Authors:** Zoë Robaey, Shannon L. Spruit, Ibo van de Poel

**Affiliations:** 1 Department of Biotechnology and Society, Delft University of Technology, Van der Maasweg 9, 2629 HZ Delft, The Netherlands; 2 Department of Multi-Actor Systems, Delft University of Technology, Jaffalaan 5, 2628 BX Delft, The Netherlands; 3 Department of Values, Technology and Innovation, Delft University of Technology, Jaffalaan 5, 2628 BX Delft, The Netherlands

**Keywords:** Safe-by-Design, Moral responsibility, Uncertainty, Synthetic biology, Group decision room

## Abstract

The Safe-by-Design approach in synthetic biology holds the promise of designing the building blocks of life in an organism guided by the value of safety. This paves a new way for using biotechnologies safely. However, the Safe-by-Design approach moves the bulk of the responsibility for safety to the actors in the research and development phase. Also, it assumes that safety can be defined and understood by all stakeholders in the same way. These assumptions are problematic and might actually undermine safety. This research explores these assumptions through the use of a Group Decision Room. In this set up, anonymous and non-anonymous deliberation methods are used for different stakeholders to exchange views. During the session, a potential synthetic biology application is used as a case for investigation: the Food Warden, a biosensor contained in meat packaging for indicating the freshness of meat. Participants discuss what potential issues might arise, how responsibilities should be distributed in a forward-looking way, who is to blame if something would go wrong. They are also asked what safety and responsibility mean at different phases, and for different stakeholders. The results of the session are not generalizable, but provide valuable insights. Issues of safety cannot all be taken care of in the R&D phase. Also, when things go wrong, there are proximal and distal causes to consider. In addition, capacities of actors play an important role in defining their responsibilities. Last but not least, this research provides a new perspective on the role of instruction manuals in achieving safety.

## Introduction

Synthetic biology (SynBio) is an emerging field that combines principles of the life sciences together with principles of engineering disciplines. The hybrid and new character of this field presents great opportunities for beneficial applications but also potentially great challenges in terms of risks and uncertainties. One of the great promises of SynBio applications is that organisms can be designed for specific functions. In addition, they can also be designed for safety by using only the necessary elements of a genome to achieve a goal, this is explained in more detail later. In this paper, some of the challenges of Safe-by-Design in SynBio and the implications for the distribution of moral responsibility are investigated. This, in turn, allows us to test some of the assumptions in the Safe-by-Design approach about distributing responsibility for dealing with risks.

The Safe-by-Design approach, as a way to deal with the potential risks of SynBio applications, seems to come with specific assumptions about which actors are responsible for properly addressing the risks of these technologies. In particular, it seems to assume that the actors in the research and development (R&D) phase bear special responsibilities for safety. This raises a number of issues. First, it raises the issue of whether all the safety issues can be taken up in the R&D phase, where design decisions are made. Second, are other stakeholders also inclined to allocate the responsibility for dealing with safety to the R&D phase? Third, one might also wonder what happens when something goes wrong, are the actors in the R&D phase blamed or does blame primarily apply to other actors in the chain from product to consumer? Last but not least, is safety an issue that can and should be left only to the actors in the R&D phase?

In order to explore these questions, a workshop was organized using the format of a Group Decision Room (GDR) session to examine the Food Warden, a biosensor contained in meat packaging that indicates the freshness of meat. This application is an innovation to the commonly used expiry date. Expiry dates on food products are often conservative for safety reasons and might be inaccurate in cases, for instance when meat is not stored properly. In this paper, a brief introduction to risk and safety issues in SynBio is given as well as to the relevant scholarship on the relationship between technological design and moral responsibility. The Food Warden case is then presented, followed by the method and the main results of this exploratory session. Finally, the findings are discussed with respect to four main points: the types of safety issues raised by participants, the concentration of forward-looking moral responsibilities in the R&D phase, the relation between forward and backward-looking moral responsibility, the forward-looking moral responsibilities of owners, and the importance of the responsible transfer of the biosensor.

## Risk and Safety in Synthetic Biology Applications

In this paper, a SynBio application that is Safe-by-Design is investigated. The claim that we can design technologies to be Safe-by-Design is not unique to synthetic biology. It can also be found in more traditional fields of engineering.

In engineering, the notion of safety has been developed in relation to the notion of risk. Safety is often defined as the absence of risk. Doorn and Hansson (2010) argue, however, that the notion of risk itself is not that clear. There are several definitions of this concept, most of which are based on a capacity to assign probabilities to certain hazards. However, in practice most hazards are not completely predictable, there are also uncertainties to take into account. In addition, one might add to their argument that, in practice what is considered as safe will vary according to perceptions of risks and safety. So there seems to be no absolute definition of what is safe.

Following these observations, Doorn and Hansson (2010) argue that to design for safety does not only imply taking into account known risks but also designing for uncertainties and hazards that are not yet known (for example by using safety factors that can also deal with uncertainties). When it comes to risk and uncertainties in SynBio, the paradigms are different from traditional biotechnology. Indeed, through SynBio, a number of engineering principles are translated from other engineering disciplines to synthetic biology. For instance many parallels are drawn with computer systems engineering or information technology systems in general (Andrianantoandro et al. 2006). To put it simply, SynBio allows the creation of biological machines. The promise of SynBio is that these machines can do whatever the designer wants them to do. In other words, SynBio simplifies the bacteria’s genome in order to keep the only preferable parts of the DNA that matter for the expression of a function. This, in itself, is already a design principle particular to synthetic biology as opposed to traditional biotechnologies. In this line of thinking, the simplification of the genetic material could play a role in increasing the safety of synthetic biology applications.

In addition, switches can be introduced in the coding of the DNA of the cell so that they can be turned on and off depending on the substrate. The possibility of having a kill switch that will disintegrate the cell membrane is also seen as an advantage in designing for safety. These are not technological innovations limited to the field of SynBio. However, in the SynBio world, there is a quest to create standardized bio bricks that could easily be assembled in any order to create whatever biological machine is required. When thinking about safety in sybio, Schmidt (2008) writes that there are different levels to consider: the individual parts, the circuits and the chassis. (Andrianantoandro et al. 2006) point to the situation that the behaviour of even simple engineered cells will be difficult to predict. Indeed, as opposed to computer systems, reliability and predictability in SynBio are problematic because single cells might behave differently than groups of cells, hence the need to consider the different states in which the cells are held.

It is difficult to speak about the risks of synthetic biology in general, as every application will have different implications, and the risks of a single application might not be as well understood as we might think. Generally speaking, the risks would be that a SynBio application does not do what it is supposed to do and instead does something else that negatively affects other things. All the promises of safe biological machines that SynBio brings, warrant an investigation of the understanding of safety for these applications.

## Design and Moral Responsibility

In engineering ethics, discussions on the relation between humans and technological artefacts are central. There is one seminal argument made by Winner (1980) which emphasizes that the design of artefacts contains choices that humans make. Therefore, they will mirror our values, intentions and decisions. (Van de Poel et al. 2014) argue that if artefacts can embody values it also means that they can be designed for values, so it is possible to design for safety. In addition, values can be translated to norms, which in turn can be translated into design requirements (Van de Poel 2013a).

However, there are fields where Safe-by-Design is more advanced and defined such as civil engineering. A straight-forward example of this is the choice of building materials to minimize the risks of fire. The field of SynBio is still in its infancy and so is its understanding of safety. There are possibilities to have safety mechanisms in the design, but it is not clear who has the responsibility to decide what safety means and how it should be implemented. This is why some clarifications are needed on both the definition of safety and on the distribution of moral responsibilities (see Doorn & Van de Poel 2012).

When looking at moral responsibility, there are two important distinctions: forward-looking and backward looking moral responsibility (e.g. Dworkin 1981; Van de Poel et al. 2015). Forward-looking moral responsibility is also called active moral responsibility and the idea that people are responsible to see to it that a certain state of affair is realized. So it is a proactive notion of responsibility. Backward-looking moral responsibility refers to responsibility after the fact. Typically in engineering ethics, there are five conditions stated to establish whether there is backward-looking moral responsibility: freedom of action, wrong-doing, foreseeability (knowledge), capacity, and causality. Examples of backward-looking responsibility are accountability (the obligation to account for an action or outcome), blameworthiness (being blamed for an action or outcome) and liability (the obligation to repair or compensate for an undesirable outcome) (see Davis 2012). In case of desirable outcomes, backward-looking responsibility may also include praiseworthiness (see Doorn 2012b).

When distributing and allocating moral responsibility the reasons to do so are important. Allocating responsibility with the goal of achieving safety can be done in different ways. A suggestion is, for instance, based on the fairness and efficacy way (Nihlén Fahlquist 2006). In addition, taking individual considerations as to how to ascribe responsibilities is important (Coeckelbergh 2012) otherwise, a few actors may be overburdened and responsibility gaps may appear, which could lead to not achieving the value at hand, in our case safety. It is also important to address how responsibilities are distributed, since actors may understand their own responsibilities and the responsibilities of others differently (Shelley-Egan and Bowman 2015).

Allocating forward-looking moral responsibilities is therefore particularly crucial when thinking of Safe-by-Design. Indeed, the idea of Safe-by-Design builds on the forward-looking moral responsibilities of designers. In that framework, designers think about safety issues and think about how their design can prevent harm. However, there is uncertainty about the real use, and impact of new technologies on societies and environment. There are impacts that can only be discovered once a technology is introduced in the real world, so even if it were to be designed for safety, there must be continuous learning after the R&D phase which cannot be designed in beforehand (Wetmore 2008). In addition, Safe-by-Design does not inform the discussion on backward-looking moral responsibility. This does not mean that the whole endeavour is pointless. To the contrary, it means that these issues of allocation and distribution expand beyond one type of actor and can involve several actors.

This is what the concept of the social experiment articulates. On the one hand, it deals with all the actors involved with the introduction of new technologies in societies and on the other hand, it deals specifically with uncertainties and how to deal with them in an ethical way (Van de Poel 2011b, 2012, 2013b; Jacobs et al., 2010; Doorn et al. 2016). Indeed, the introduction of new technologies can bring great benefits but also potential hazards, and it can be understood as a social experiment. This proposal encompasses questions of distribution and allocation of moral responsibilities, forward, and backward, as well as strategies to deal with the introduction of new technologies, like for instance through learning. In the social experiment, all actors can be considered experimenters; it provides a new dimension to the governance of risks and uncertainties.

Building on the notion of the social experiment, another recent proposal suggests allocating forward-looking moral responsibilities to owners of technologies. The reasoning behind this proposal is that since owners reap benefits, they also bear special forward-looking responsibilities for the technologies they own. These responsibilities could include learning about these technologies in order to be able to act when unknown risks would start materializing (Robaey 2014, 2016).

In a nutshell, the social experiment, the responsibility of owners, and the responsibility to learn about unknown risks can provide a useful analytical lens to study the distribution of moral responsibilities in Safe-by-Design for SynBio applications. Asking whether all safety issues can be taken up in the R&D phase, and whether other actors agree with this, are questions of allocation and distribution of forward-looking moral responsibilities. Looking at backward looking moral responsibility addresses questions of who is to blame when things go wrong. Taking a broader view at safety beyond Safe-by-Design, by looking through the lens of the social experiment, allows thinking about what safety and responsibility mean for the case at hand.

## Case: The Food Warden

In order to carry out a discussion on the distribution of moral responsibility for Safe-by-Design in synthetic biology, we wanted to choose an exemplary case of SynBio. However, there are a few SynBio applications currently being used, but they are all applications that are in a contained environment, such as the production of artemisin by Amyris^®^, of vanillin by Evolva^®^, and of algal oil by Ecover^®^ (cf. Aveld and Stermerding 2016). To carry out a fruitful discussion the case required certain characteristics, but especially be an exemplar of a difficult containment and easy dispersion of the SynBio application in order to broaden the scope of stakeholders. The chosen case is therefore a potential SynBio application that could exist but does not exist yet. This provided the advantage to not be constrained by the reality of an existing case, but also the disadvantage was that it was more difficult to motivate stakeholders to consider a case that does not yet have a clearly defined community.

Bearing this in mind, the case chosen was developed by the 2012 team from the University of Groningen for the International Genetically Engineered Machine Competition (iGEM), the Food Warden, a concept for a biosensor. The biosensor is to be placed in a resistant pocket as part of the packaging and would replace the expiry date by detecting, or “smelling” when meat goes rotten. The team argues that using this technology would be safer and more accurate than the currently used expiry date system. Also, using this technology would address a major societal problem of food waste by providing more accurate information on the freshness of meat.

In terms of Safe-by-Design, there are several elements that make the bio-sensor safe (iGEM Groningen 2012). The bio-sensor is (1) made from soil bacteria that are harmless to humans, it is (2) contained in a solid packaging that is ostensibly unbreakable, and it has (3) a limited nutritional substrate, which is also the only substrate in which it can survive.

While there is no present implementation of the concept, the team had gone far in the development of a prototype, and won the Gold Medal of the iGEM competition that year. For the purpose of this research, a future where the Food Warden is being used in the market is assumed.

## Methods

### The Group Decision Room

In order to explore the questions on moral responsibility and Safe-by-Design, a workshop under the form of a Group Decision Room (GDR) session was organized. The goal of a GDR session is to solve complex issues in a collaborative manner using virtual communication tools (cf. Kolfschoten and de Vreede 2009).

The use of virtual tools helps preserving the anonymity of participants During a GDR session participants each take place behind their own laptop in a room. Individual questions are interchanged with plenary presentations and discussion. This GDR session, that took place on September 30, 2015, included anonymous discussions via *Meeting Sphere*, opinion survey via *Lime Survey* and open discussions that were observed and recorded by means of notes by the researchers. The facilitation was the result of collaboration between the researchers and a professional facilitator.

These tools allow carrying an anonymous but lively written discussion, as well as presenting and organizing the information in an almost real-time fashion, and giving and receiving feedback on the discussion. In the GDR, people’s opinions on different issues are collected and their assumptions and reasoning are challenged by going over an issue in different ways and by providing input, or new perspectives that help participants form an opinion. The general method for this GDR is inspired by Doorn (2010, 2012a).

### Structure of the Session

In order to gain insights on the relation of Safe-by-Design and moral responsibility in the Food Warden case, the session was divided into five main steps (see Table 1). After the introduction of the case, a brainstorm with the participants allowed to identify issues of concern. Then, these issues were used to investigate the distribution of forward-looking moral responsibility and a scenario for distribution of backward-looking moral responsibility (see Table 5). As a last and somewhat separate exercise, the distribution of ownership rights was examined. Before starting the introduction the participants were assigned an anonymized alter ego for the GDR software. Each step had presentations, online and offline discussions, as well as votes on different issues. This programme had been tested out and adjusted based on the experience of a group of civil servants, before running the GDR session on which this paper is based.

**Table 1:** Protocole

Programme part	Time (approximation)	What
Pre-workshop		Allocation of alter-ego for the session by observer
1. Introduction	15 min	Introductory round participants and facilitators Explanation goals GDR session Explanation software Informed consent form reading and signing
2. Identification issues of concern	45 min	Presentation developer on Foodwarden Presentation facilitator on Safe-by-Design Brainstorming safety issues via Meeting Sphere Individual voting on two most import and one ‘new’ safety issues via meeting sphere Plenary ordering of safety issues based on voting
BREAK	10 min	
3. Distribution of forward-looking moral responsibility	1 h	Explanation phases by facilitator (see next subsection for explanation phases) Participants are asked to individually connect issues to one or more phases and explain their choices via LimeSurvey Voting results are shown, followed by a plenary discussion on the results Second round of voting on the same topic via LimeSurvey Participants are asked to allocate themselves to a phase and explain why
BREAK	10 min	
4. Distribution of backward-looking moral responsibility	1 h 10 min	Presentation on the catastrophe scenario developed by the Dutch Institute for Health and the Environment Discussion in *Meetsphere* where participats are asked to indicate who would blame whom (a blame game) Participants vote in *LimeSurvey* who is responsible in catastrophy scenario, and explain their choice Presentation by facilitator on the relation between knowledge and moral responsibility Participants individually vote in *LimeSurvey* on the level or knowledge stakeholders in each phase should have had
BREAK	10 min	
5. Distribution of ownership rights	10 min	Presentation on ownership rights Individual vote on the distribution of ownership rights to stakeholders via LimeSurvey
Round up	15 min	Plenary evaluation

### Distinction of Phases

As part of the preparatory work for the GDR (used for part 3 of the programme), the development and use of the Food Warden was divided into six phases (see Table 2). These phases are identified in the tradition of science and technology studies that suggest following the object (cf. Latour 1987) and trace the journey of the biosensor from the its conception to its disposal. This was important in the GDR session because they allowed creating a proxy to talk about in which phase responsibilities are located instead of pointing to stakeholders who would tend to have these responsibilities. Defining the phases prior to the session also allowed mapping the relevant stakeholders.

**Table 2:** Phases and their main actors

Phases/stakeholders	Research and development	Market approval	Use in Industry	Retailer	With the consumer	Waste disposal
	Scientists (universities, companies); Companies in material, packaging, SynBio	I&M; RIVM; Ministry of Health; Ministry of Economic Affairs	Meat industry; packaging industry	Different supermarkets; restaurants	Consumers; consumer watchdog	Waste removal companies; municipality; health inspection

### Participants

Participants relevant to each phase were invited. In the end, 11 participants from industry, government and science, were present at the session (see Table 3 for an overview). Unfortunately, none of the civil society invitees participated in the workshop, which may result in the position of consumers being less well defended. The number of participants underlines the exploratory nature of this research and stresses that the results presented are only relevant to this case and might provide avenues of inquiry in other cases, as will be examined in the discussion.

**Table 3:** Participants’ sectors

Code	Sector
Q45	National government
H11	National government
A12	National government
S73	National government
C31	Regulatory organisation
W19	Research organisation
F06	Research organisation
A08	Developer
R36	Meat industry
K55	Packaging industry
Z04	Packaging industry

## Results

Before being able to speak of the distribution of moral responsibilities, the most important part of the session was to identify the issues of concern. After the presentation of the Food Warden participants were asked to identify potential safety issues of this product by answering the question, “what could go wrong with the biosensor?” Table 4 gives an overview of the issues that were selected for further discussion because they were (1–8) deemed important by the group, and (9–11) considered surprising.

**Table 4:** Issues of concern for the biosensor

Issues
(1) The bacteria will adapt in an undesirable way
(2) Misuse
(3) Illusion of safety (perception of the consumer)
(4) The bacteria survives outside the packaging
(5) Sensitivity is not appropriate
(6) Fraud
(7) The sensor is unreliable
(8) The bacteria comes in contact with meat
(9) The sensor is ingested by a child
(10) Transfer of genetic traits to nature
(11) The instructions for usage are unclear

These issues were originally listed in Dutch and translated to English by the authors

A striking observation is that while participants were asked specifically about the safety of the Food Warden itself, some of their answers seem to go beyond the purely safety issues of the product. Fraud (issue 6) and problems of misuse (issue 2 and 9) do not have to do with some inherent feature of the Food Warden itself, but rather identify negligence or malicious intent as causes for unsafe practices. Issues 3 and 7 seem to hint at how the introduction of a technology such as Food Warden influences our perception of safety, and show that a conflict between our perception of safety and actual safety in itself poses a safety risk.

**Fig. 1:**
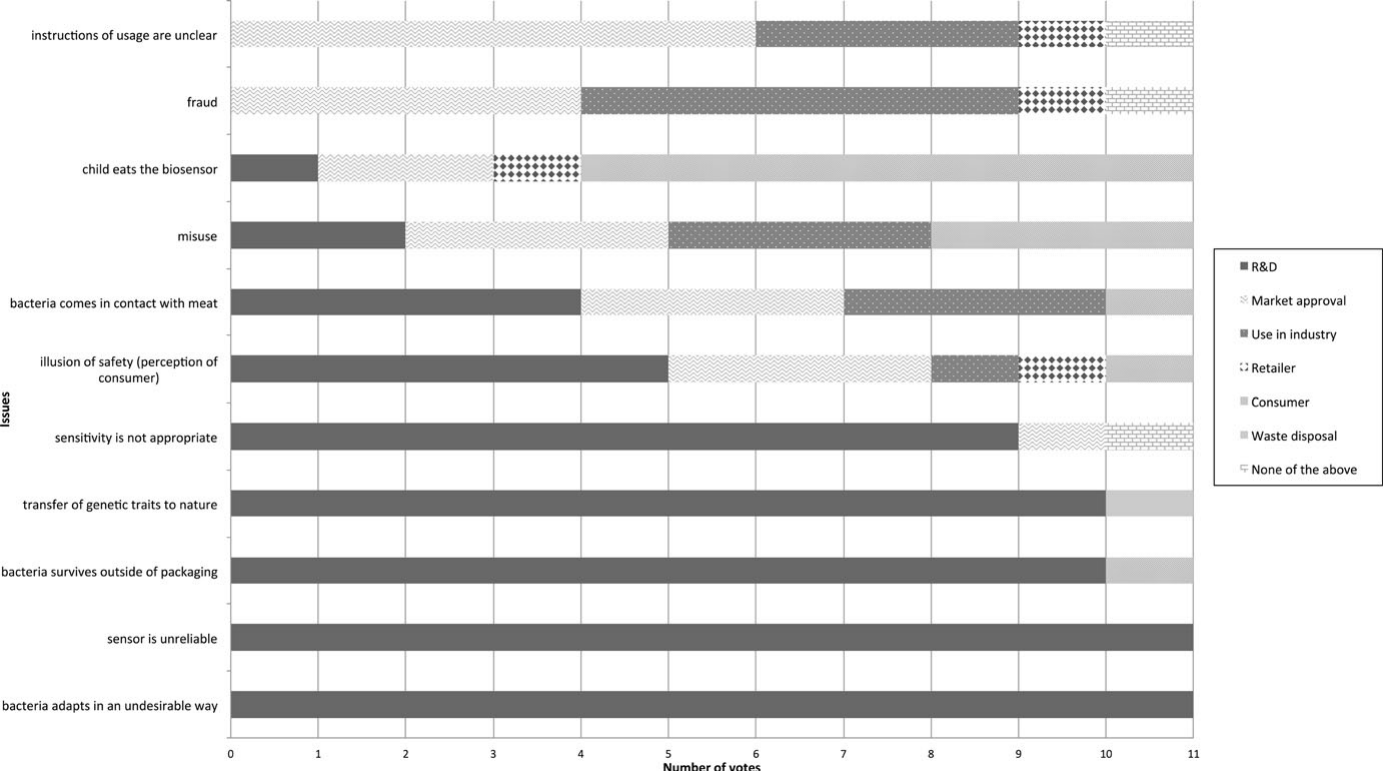
Results of Vote 3: participants allocate each issue to only one phase

Once these issues were listed, three rounds of voting were carried out for participants to allocate these issues to phases. The multiple rounds of voting allowed the participants to become more familiar with the issues and clarify differences in interpretations along the way. In the first round, participants were able to allocate issues to several phases. In the second round, they could allocate issues to only one phase. The second round was followed by a discussion of the results of that vote (an excerpt is included in the discussion section), and it was followed by a third and last round of voting, where again, each issue could only be allocated to one phase (Fig. 1).

After these votes, a problem scenario was presented, where many children got ill at a party after eating meat that was packaged using the Food Warden and used by a snack bar for burgers, i.e. the retailer who improperly made burgers that were toxic (see Table 5).

**Table 5:** The catastrophe scenario

During a children’s party, all kids got ill after eating hamburgers from a snack bar. After angry phone calls from worried parents, the snack bar owner realizes that some of the Food Warden pockets are ripped open. The hamburgers were purchased at a retailer that uses the Food Warden as an indicator of freshness. It is unclear when those pockets containing the Food Warden might have broken open. Fortunately, all the children recover very quickly. However, the parents refuse to go eat at establishments that make use of the biosensor and they share their outrage on social media	

**Fig. 2:**
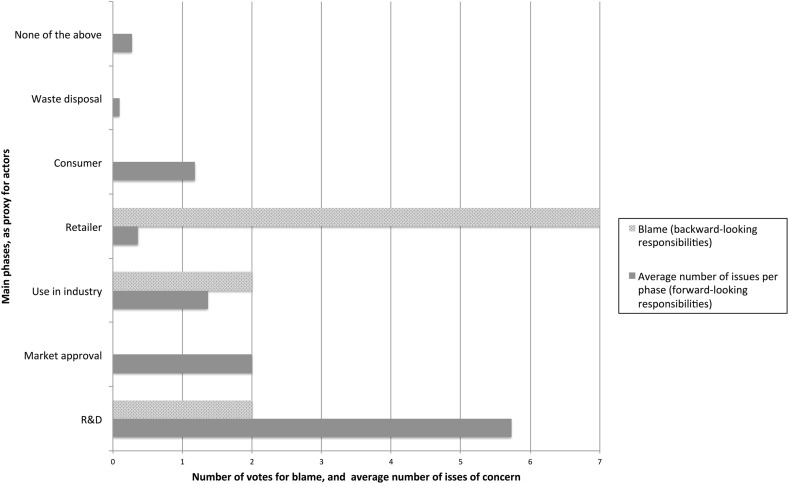
Comparison of backward-looking responsibility versus forward-looking responsibilities

Participants had to vote for those they thought would be to blame for the children getting sick. In Fig. 2, these results are compared with the “amount” (i.e. the average number of issues that were allocated to phases in vote 3) of forward-looking responsibilities that were allocated to the same phases.

**Fig. 3:**
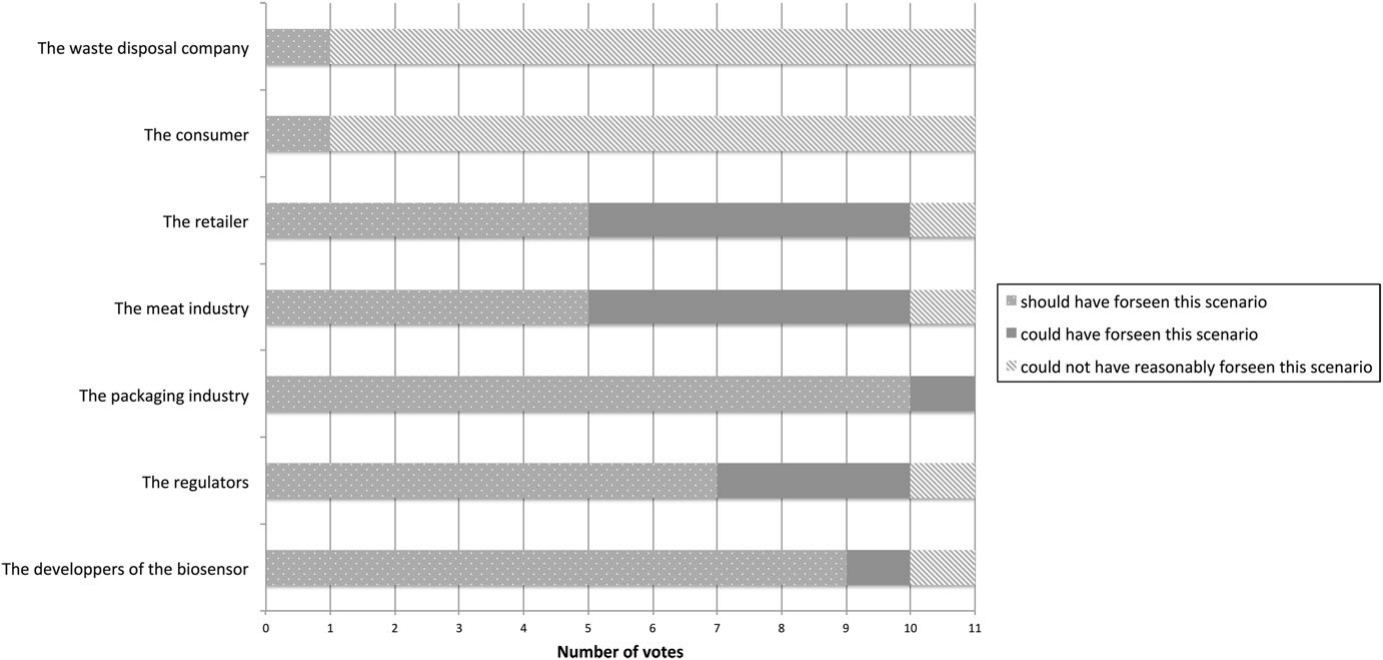
Votes on foreseeability in the catastrophe scenario

After this vote, we asked participants to reflect on the knowledge condition of backward-looking moral responsibility. This will be expanded upon in the discussion, but for now Fig. 3 depicts is what participants answered.

The issue of backward-looking moral responsibility was further discussed offline, and online through an anonymous discussion where participants had to put themselves in the shoes of other stakeholders. These results are not included here as they were not the most salient for the issues under consideration but some observations by participants do come back in the discussion.

As a last step, participants’ perceptions on ownership rights were explored. Participants were asked to ascribe the different stakeholder the ownership rights they thought these stakeholders should have. Table 6 shows their votes compared with the number of issues per phase at vote 3. Only the results of the third vote is used, as we understand them as the most stable agreement.

**Table 6:** Votes for ownership rights allocated to actors in phases compared to number of issues per phase from Vote 3 (in italic)

Stakeholder/rights	R&D	Market approval	Use in packaging industry	Use in the meat industry	Retailer	Consumer	Waste disposal
Right to use	7	2	6	9	7	8	2
Right to manage	6	5	5	3	0	0	0
Right to transfer	11	0	3	3	2	1	1
Right to income	11	1	8	5	4	0	2
*Number of Issues (vote 3)*	*9*	*7*	*5*	5*	*4*	*5*	*1*

* Idem for both industries

## Discussion

In the introduction, the concept of Safe-by-Design was presented as one that shifted most of the burdens of moral responsibility to the actors in the R&D phase. As this was an exploratory research, it started with a number of questions that are answered in this discussion, but new questions were also formulated from our findings. In the case of the Food Warden, can the actors in the R&D phase take up this responsibility, and should they? Who bears what forward-looking moral and backward-looking moral responsibilities? Do owners bear special responsibilities? Finally, how are responsibilities transferred? Our exploration of the case does not provide generalizable evidence for all SynBio applications, but a number of interesting things are observed.

### On the Concentration of Moral Responsibility in the R&D Phase

First, Table 1 shows that at the 3rd round of voting, and after discussions, participants felt that five of 11 issues were in the R&D phase (all participants or a clear majority of participants). These five issues of sensitivity and reliability, the possibility of the bacteria adapting in an undesirable way and surviving outside the packaging, or even transferring genetic traits to other organisms in nature. In other words, participants identified these issues and then decided that the actors who had the capacity to prevent those issues engaged were in the R&D phase. This is far from surprising as all these issues concern the design of the Food Warden.

However, some participants allocated other issues to the R&D phase, albeit not by a majority. These issues are that the bacteria come in contact with meat, that a child eats the Food Warden, the possibility of misuse and the illusion of safety. This could be interpreted as that although actors in the R&D phase do not necessarily have these forward-looking moral responsibilities, some of the participants believed that they might have the possibility to influence the likeliness of these issues.

All in all, actors in the R&D phase do seem to be bear the most forward-looking responsibilities according to our participants, supporting what one would expect with the use of a Safe-by-Design approach. It should be noted, however, that participants from the R&D phase were under-represented in our GDR session. Further research might want to focus on whether these actors are willing and able to undertake these responsibilities. Moreover, although the only participant that is actually working in the R&D phase allocated a lot of the issues to the R&D phase, she also allocated several issues to the waste disposal phase, a phase that was not paid attention to by other participants. Also, with the experience of responsibility ascription with nano-materials (cf. Shelley-Egan and Bowman), it is important to bear in mind that different stakeholders might understand their own, as well as others’ responsibilities differently.

### On the Relation between Forward and Backward-looking Moral Responsibility

Now that we have established that in our GDR session, many of the forward-looking responsibilities are allocated to the R&D phase, it is time to look at whether this is the case for backward-looking moral responsibility. Before investigating whether there was any relation between how the participants allocated forward-looking and backward-looking responsibility, an explanation is needed as to why one might expect such a relation to exist, at least philosophically.

According to most philosophical theories of responsibility, the appropriate apportioning of backward-looking responsibility (like accountability, blameworthiness and liability) depends on a number of conditions. One of the conditions that is regularly mentioned in the philosophical literature for backward-looking responsibility is wrong-doing. One cannot be blameworthy for an action or outcome if one did nothing wrong. Elsewhere, it has been argued that wrong-doing might either involve not living by a forward-looking responsibility or the breaking of a duty (Van de Poel 2011a, b). According to Goodin (1986) duties are the deontological pendant to a consequentialist definition of responsibility. In other words, a duty prescribes a defined action, while a responsibility prescribes a desired outcome. Not fulfilling a duty, or failing to achieve a desired outcome can both be seen as the breaking of a moral obligation.

One might expect that for most people, like the participants in our sessions, the distinction between breaking a duty and not living by a forward-looking responsibility is too fine-grained to make a real distinction in their reasoning. Therefore, our participants were expected to see all moral obligations as forward-looking responsibilities and that there would be a certain relation between how they attributed the forward-looking responsibility and how they attributed backward-looking responsibility. However, Fig. 2 suggests that such a relation was absent. It should be noted that the retailer in Fig. 2 corresponds to the snack bar in the scenario, as was explained to the session participants.

One possible reason why Fig. 2 does not show a relation between forward-looking and backward-looking responsibility is that the attribution of backward-looking responsibility is based on the scenario presented above, while the forward-looking responsibilities relate to a range of possible risks. The scenario was deliberately formulated broadly and somewhat ambiguously so that it would not point at one cause but at a range of possible causes for the sickness of the children. When looking at the various safety issues inventoried (see Table 4), it would seem that all of these except for one could be among the causes of the sickness of the children in the scenario. The only risk that would seem impossible as the cause in the specific scenario is the risk of gene transfer to the environment. Nevertheless, it is conceivable that the participants when interpreting the scenario ruled out possible safety issues as causes. This seems indeed the case as some participants in part 4 of the GDR gave as explanations for attributing the responsibility to the retailer that the Food Warden has been tested and therefore had no influence on the meat.

It should be noted that the above highlights a more general difference between attributing forward-looking and backward-looking responsibility. In attributing backward-looking responsibility, one specific event that has already occurred is usually considered, while in the case of forward-looking responsibilities a much larger range of possible scenarios that could occur but need not have occurred yet are considered.

Another possible reason why Fig. 2 does not show a relation between forward-looking and backward-looking responsibility is that wrong-doing is only one of the conditions for backward-looking responsibility. Another condition is knowledge, or more precisely the ability to foresee certain scenarios or consequences. Participants were therefore also asked whether according to them the various actors should have foreseen this specific scenario (see Fig. 3)

However, it would seem that the results as presented in Fig. 3 do not explain why most participants attributed responsibility to the retailer as the participants apparently did not believe the retailer to be in the best position to foresee this scenario. Moreover, it also does not explain why they attributed quite a lot of forward-looking responsibility to the actors in the R&D phase but much less backward-looking responsibility to these actors as 9 out of 11 of the participants voted that the actors in the R&D phase should have foreseen this scenario.

To understand why most of the participants attributed backward-looking responsibility to the retailer, it is worthwhile looking at the justifications they gave for this attribution in part 4. Two things are outstanding. First, some participants state that the retailer is blame-responsible until it can be proven that a mistake has been made elsewhere in the causal chain. A second remarkable argument is that it is the first in the causal chain that should have the blame from the viewpoint of the consumer.

The first argument is interesting because it suggests a heuristic in attributing blame-responsibility (‘blameworthy until proven innocent’) that seems to be the opposite of the legal rubric that someone is innocent until proven guilty. This heuristics is perhaps less amazing as it seems because also in many real-world cases of disasters or scandals people feel that somebody should be blameworthy, even if there are reasons to believe that there might be situations in which no one can reasonably be blame (like in cases of the problem of many hands, see for example Van de Poel et al. (2012).

This heuristic may also be related to the so-called Knobe-effect. Knobe (2003) found experimentally that in cases of undesirable outcomes, intentionality or blameworthiness is more likely to be attributed to the agents causing the undesirable outcomes than in cases of good or desirable outcomes. This may possibly explain why the retailer is only attributed with limited forward-looking responsibility but much more backward-looking responsibility after the unfolding of a scenario with an undesirable outcome.

However, this would not seem to explain why the participants in the majority hold the retailer blame-responsible rather than for example the developers of the Food Warden. One possible explanation here is the distinction between proximate and distal causes. If something undesirable happens it typically does not have one but many causes (Del Frate et al. 2011, Bhaumik 2009). The proximate causes are the causes that are most direct and attract initial attention. In this specific case, the quality of meat that was delivered by the retailer is likely to be a proximate cause of the illness of the children. In contrast to proximate causes, distal causes or the so-called root cause lie earlier in the causal chain, but often are considered more important or fundamental for avoiding certain scenarios. One might hypothesize that in attributing forward-looking responsibility, people tend to focus on distal causes as they are often more important in avoiding certain scenarios. While once something undesirable has happened, people may well focus on more proximate causes, especially if there is limited information available about the scenario that has unfolded or if the scenario is ambiguous (as is often the cases in real-life situations and also in the scenario that we presented). Such a hypothesis would explain the observations in this research, in particular the disconnection between the attribution of forward-looking and backward-looking responsibility.

### On the Forward-looking Moral Responsibilities of Owners

In the introduction, the implication that Safe-by-Design meant that only actors in the R&D phases would bear moral responsibilities for the safety issues of the Food Warden was questioned. A proposal for allocating forward-looking moral responsibility to owners, so not only designers, of that technology was briefly presented.

The idea behind this type of forward-looking moral responsibility ascription is that if owners reap the benefits of a technology, then they should also have forward-looking moral responsibilities to do no harm with that said technology (Robaey 2014). This concerns new technologies with potentially great benefits but that also entail uncertainties and unknowns, when the introduction of a new technology in society can be considered a social experiment (Van de Poel 2011b).

How can owners avoid harm, if there are many uncertainties and unknowns that come with the use of a technology? Robaey (2016) argues that ignorance does not absolve responsibilities, so if owners do not know the possible hazards of their technology, they ought to learn about them. The way owners learn, i.e. what they will learn about and how, will depend on the cultivation of their epistemic virtues and their capacities. Owners have to act as responsible experimenters, or in other words, to learn about these technologies, in case that they might have unintended side effects, so they can react.

Ownership is here conceived of as a bundle of rights and responsibilities (Honoré 1961). With regard to rights, different owners can have varying amounts of rights over a technology, what Honoré calls split ownership e.g. only the right to use and the right to income, or only the right to manage. But every owner of a technology, or its copies, has the responsibility to do no harm (or prevent it). In turn, this responsibility is translated for each owner into a *range of actions*, that are themselves defined by capacities and contexts.

At the start of the GDR session, potential issues with the Food Warden were identified. These issues can also be understood as the basis of defining a range of actions that stakeholders (of which some are owners) can take in order to avoid undesirable outcomes.

Indeed, the issues listed are all connected to the responsibility to do no harm, and also, in a more or less direct manner, to learning about potential effects, or rather learning how to deal with these potential issues. Also, for the purpose of this exercise, only ownership rights that were most relevant to the case at hand were considered: the right to use, the right to manage, the right to transfer, and the right to income. These were most relevant because of the type of issues we could expect with biosensor SynBio application such as the Food Warden that would have to do with containment, potential malfunctions and issues in use.

An initial assessment of the relationship between ownership rights and moral responsibility could make for the following hypothesis: the more rights owners have over a technology, the more they should have responsibilities. However, as Robaey (2016) points out, an important criteria for responsibility is the owners’ capacities rather than their rights (although more rights might provide for more capacities). In this explorative set-up, the question of ownership was not at the centre of investigation. Also, explanations were kept to a minimum regarding the rights; this might have led to a varying range of interpretations by the participants. However, some initial results were gathered that can provide a base for discussing the insights above.

In Table 6, for the actors involved in the R&D phase, it seems that participants generally ascribed to the owners all of the rights with a majority of the votes. Also, most of the issues are allocated to the R&D phase. It corresponds to the idea that the more ownership rights actors may have, the more forward-looking responsibilities they would have. A similar but less pronounced pattern is observed in the industrial phases.

There are two phases that stand out particularly when considering the relationship between rights and responsibilities: the market approval one, and the consumer one. In both these phases, the hypothesis that more rights entail more responsibilities does not hold. Indeed, for both phases there are overall lesser rights, but a high proportion of forward-looking moral responsibilities. For instance, the right to manage received many votes at the market approval phase (i.e. the regulators), but not the other rights. Likewise, the right to use for the consumers received many votes, but not the other rights. These two groups of actors are responsible for a large number of issues overall but have less rights overall.

During the GDR session, there might have been a misunderstanding of the meaning of ownership rights. It is very well possible that participants interpreted the “right to” of an actor, as an actor “can do/have X”. This interpretation would support the idea that the actual allocation of forward-looking moral responsibility is more strongly connected to the idea of capacity, i.e. what actors can do. Therefore, there does not seem to be a straightforward relation between the allocation of forward-looking moral responsibilities and the allocation of ownership rights.

When looking at the issues that participants listed, that are not strictly linked to the design of the Food Warden, it becomes clear that these tend to be split between different phases (Fig. 1), like with misuse, fraud, unclear instructions, a child eating the Food Warden, and the illusion of safety. This could indicate that participants focussed on different capacities of actors in each of the phases to do something about the issues.^1^

Ascribing forward-looking moral responsibilities to owners should allow them to define their range of actions to do no harm when acquiring the Food Warden. These actions depend on their capacities, and also do not exclude other actors from taking responsibility; it only puts the emphasis on forward-looking responsibilities of owners that have been left unexplored. Making these explicit can only enhance the chances of an appropriate use of new technologies, such as the Food Warden. So Safe-by-Design also involves other actors than the ones in the R&D phase. In the next section, an unexpected observation is presented that goes on to specify a claim for broadening the scope of actors.

### On the Importance of Responsibility Transfer

As presented in the results, the issues that were raised did not all pertain strictly to the idea of Safe-by-Design. Indeed, several of the issues raised are linked to the use and social context of the Food Warden. In this section, special attention is payed to the transfer of responsibility through instruction manuals, which appeared in the session as an issue (issue 11, ‘instructions for usage are unclear’) and around the discussion on misuse.

Before participants were asked to allocate issues to one phase, there was a plenary discussion on the answers collected until then. The following is an excerpt of the discussion that followed after the facilitator asked why participants had allocated the issue of misuse to a specific phase.^2^ A participant from the national government (Q45) starts of by explaining why she chose to allocate the issue misuse to the phase of market approval.

Q45: Misuse should be listed in the manual, this should be checked during market approval.

F06: But you often see that people don’t do this.

Q45: That’s why you have to inform the consumer, for instance through [organization removed for anonymity]^3^

[Unidentified participant]: Maybe the consumer should do this herself.

A12: explains that irrespective of the manual the responsibility for misuse is with the consumer.

R36: But this does not absolve producers from their duty to put a good product on the market.

The discussion concerning the creation of a false sense of security links up to this topic as well:

Q45: This is analogous to our earlier discussion on the manual. This [Food Warden] is a way to indicate the shelf life of meat. But it includes uncertainties as well, so you should indicate under what circumstances it can be used; ‘No not in such and such cases’ and ‘Yes, if…’.

W19: ‘when in doubt…’

R36: You could also make a claim based on science.

K55: What kind of manuals do you expect? I wouldn’t expect more than one sentence.

Facilitator: That’s a good question.

F06: You should indicate the circumstances under which it works.

[Unidentified participant]: That would be a reason to go back to the R&D phase, to ensure it always works. To make a foolproof product.

A12: The use of the sensor [Food Warden]would be determined to a great extent. Including [the risk of] breaking it and what he sensor [Food Warden]indicates.

Z04: and that [information] fits on the sensor [Food Warden]? …

These observations allow the raising of an important question on the role of instruction manuals as a way to negotiate responsibilities. If instruction manuals are meant to prevent misuse, and thereby foster a responsible use of the Food Warden, then who should be responsible for what they contain and how should they be written?

According to the participants, votes casted after the above-discussion shed a divided outlook on the distribution of forward-looking responsibilities. Around half of participants allocated the issues of ‘the instructions are unclear’ to the market approval phase, about a quarter of participants to the use in industry, with the remaining votes split between the retailer and R&D phases. The majority of votes, slightly more than half for the issue of ‘misuse’, are split between the use in industry and the consumer phases, with the rest of the votes placed towards the R&D and market approval phases.

These results are confusing because it seems that the same actors (for the most part) are involved in defining the instructions, and misusing the Food Warden. But it is this very confusion that promotes the shedding of light on the potential that instruction manuals have in achieving the value of safety.

Instruction manuals act as a way to indicate a way of using a technology to achieve desired ends, or in other words, to use a technology safely. However, instruction manuals can also act as a way for producers to deflect liability. They present one way of using an object and if this is not followed and something or someone is damaged, then the producers are not liable. This is, however, a backward-looking understanding of moral responsibility. In this paper, a forward-looking definition of moral responsibility is under study, and how instruction manuals can contribute to increasing safety.

In the design literature, instruction manuals can be understood as a use plan (Houkes and Vermaas 2004). A use plan is a rational sequence of action that leads to the realization of a goal, as intended by the designer; it is therefore prescriptive. In reality, there could be more than one use plan, but there is one prescribed set of instructions, that might be transmitted via sentences or graphics. As a participant points out, instructions may be quite succinct, and could be as short as one sentence.

Instructions can differ greatly amongst technologies such as drugs, which have very long instructions inside the packaging and clear intake instructions from the physician and the pharmacist. Or cars, which are black-boxed to a certain extent, leaving exposed some basic component for the user to be responsible for and leaving the rest to the car mechanic. As mentioned earlier, the way instructions are formulated and presented are to prescribe use, and deflect liability.

Instructions are written to prescribe a proper use of a technology and avoid undesired events designer and developers think might happen. Regulated products and their instructions are also heavily regulated. This process of formulation leaves out two important components: what users actually do, and all the potential unknown events that might happen. It is a lot to ask of instructions to cover all eventualities imaginable and unimaginable. Also, as mentioned in the introduction, different SynBio applications may warrant different types of concerns regarding uncertainties. Dealing with a technology is therefore a dynamic process (in terms of users, and effects), and currently instruction manuals may not be the most suitable reflection of this. But what does this mean for the transfer of moral responsibility?

In this GDR session, regulators, industry, R&D personnel and retailers are all involved in the issue of ‘unclear instructions’ according to different participants. The construction of instruction manuals can therefore become a locus of negotiation between the different stakeholders. In theory, one could argue that designers have the obligation to produce effective instructions with a product. However, participants of our GDR session seem to think that not all the responsibility can remain there. Also, it is inevitable to transfer some amount of responsibility with the transfer of an object (Pols 2010), the question is therefore: how to transfer responsibility it in a desirable manner.

This opens the debate to re-think the formulation of an instruction manual as a place of negotiation for the distribution of moral responsibility. This negotiation should entail the awareness that there could be more than one way to use the Food Warden and that these might also lead to proper use. Moreover, using the notion of the social experiment as a frame to deal with the negotiation of moral responsibility broadens the scope of actors that will be involved earlier in the process, and allows actively sharing the forward-looking moral responsibilities with actors beyond the R&D phase. In a way, all actors involved in the use of the Food Warden are experimenters, and there are responsible ways to experiment. Perhaps the locus of negotiation of an instruction manual could be where various actors and stakeholders come together to define how they will experiment with the Food Warden, instead of following a linear journey of product development, approval, market placement and use.

## Conclusion

While the results of this GDR session are exploratory, they have allowed reflection on a number of issues on the theme of Safe-by-Design in SynBio. First and foremost, when presented with a Safe-by-Design SynBio application, participants do tend to put many possible safety issues into the R&D phase. Safe-by-Design seems to place most of the forward-looking moral responsibilities in the R&D phase for the Food Warden case. Does this also mean that backward-looking moral responsibility is also mostly in the R&D phase?

Somewhat surprisingly, there was no relation between the attribution of forward-looking and backward-looking responsibility. A possible reason for this may be that in attributing backward-looking responsibility people tend to focus on the proximate causes of the specific scenario that unfolded and on the actors connected to these proximate causes while in the case of forward-looking responsibility people tend to focus on distal (or root) causes.

Another finding is that not all safety issues listed by the participants are strictly connected to design and this broadens the horizons of what safety means when Safe-by-Design principles are used. From the very beginning, this investigation points to a salient assertion: Safe-by-Design does not solve all safety issues. Safe-by-Design only goes so far in terms of safety, because safety is not only established in design but also in use and misuse.

So there are forward-looking moral responsibilities to be allocated to actors in other phases. Looking at owners of technologies and their special responsibilities to learn about the technologies of which they reap benefits, underlined the importance of capacities rather than ownership rights. Further research could look into whether actors’ capacities can be understood as responsibilities to take actions that could prevent harm. This could have implications on how responsibilities are distributed.

Looking at these exploratory results through the lens of the social experiment showed that the negotiation that leads to distributing and allocating moral responsibilities could be done at the moment where instructions for use are defined by the different stakeholders.

In a nutshell, despite the promises of Safe-by-Design, safety cannot be achieved with Safe-by-Design only. However, looking at the journey of the Food Warden, interesting places where further research could be carried out are identified. For instance, what are the implications of proximal and distal causes on the ascription of responsibility? How do the capacities of non-regulatory and non-R&D actors impact ascriptions of forward-looking moral responsibility? Last but not least, how can instruction manuals become a locus of negotiation for the responsibilities of different actors in a social experiment with SynBio applications? Looking into these questions can provide for a constructive way to manage non-contained SynBio applications to achieve greater safety.

## Footnotes

1 Results of vote 1 were not presented, because what they showed what that all issues could be allocated to all phases, so in other words, following this reasoning, it would mean that everyone is responsible for everything. This could also be interpreted, as there are always capacities at some level to do something about an issue.

2 GDR observation notes (from 15:12 onwards), translated from Dutch to English by authors.

3 [Removed for anonymity].

## References

[CR1] Andrianantoandro E, Basu S, Karig DK, Weiss R (2006). Synthetic biology: New engineering rules for an emerging discipline. Molecular Systems Biology.

[CR2] Asveld L, Stemerding D (2016). Sustainability and synthetic biology: The case of ecover.

[CR3] Bhaumik SK (2009). A view on the general practice in engineering failure analysis. Journal of Failure Analysis and Prevention.

[CR4] Coeckelbergh M (2012). Moral responsibility, technology, and experiences of the tragic: From Kierkegaard to offshore engineering. Science and Engineering Ethics.

[CR5] Davis M (2012). ‘Ain’t no one here but us social forces’: Constructing the professional responsibility of engineers. Science and Engineering Ethics.

[CR6] Del Frate, L., Zwart S. D., Kroes P. A. (2011) Root cause as a u-turn.In *The fourth international conference on engineering failure analysis part 1, 18*(2), 747–758. doi:10.1016/j.engfailanal.2010.12.006.

[CR7] Doorn N (2010). A procedural approach to distributing responsibilities in R&D networks. Poiesis & Praxis.

[CR8] Doorn N (2012). Exploring responsibility rationales in research and development (R&D). Science, Technology and Human Values.

[CR9] Doorn N (2012). Responsibility ascriptions in technology development and engineering: Three perspectives. Science and Engineering Ethics.

[CR10] Doorn N, Hansson SO (2010). Should probabilistic design replace safety factors?. Philosophy & Technology.

[CR11] Doorn N, Spruit S, Robaey Z (2016). Editors’ overview: Experiments, ethics, and new technologies. Science and Engineering Ethics.

[CR12] Doorn N, van de Poel I (2012). Editors’ overview: Moral responsibility in technology and engineering. Science and Engineering Ethics.

[CR13] Dworkin G (1981). Voluntary health risks and public policy: Taking risks, assessing responsibility. Hastings Centre Report.

[CR14] Goodin RE (1986). Responsibilities. The Philosophical Quarterly.

[CR15] Honoré, T. (1961). “Ownership.” In *Oxford essays in jurisprudence: A collaborative work*, edited by A.G. Guest. Oxford University Press.

[CR16] Houkes W, Vermaas P (2004). Actions versus functions: A plea for an alternative metaphysics of artifacts. The Monist.

[CR17] iGEM Groningen 2012. n.d. “Safety.” http://2012.igem.org/Team:Groningen/Safety.

[CR18] Jacobs JF, van de Poel I, Osseweijer P (2010). Sunscreens with titanium dioxide (TiO_2_) nano-particles: A societal experiment. NanoEthics.

[CR19] Knobe J (2003). Intentional action and side effects in ordinary language. Analysis.

[CR20] Kolfschoten GL, de Vreede G-J (2009). A design approach for collaboration processes: A multimethod design science study in collaboration engineering. Journal of Management Information Systems.

[CR21] Latour B (1987). Science in action: How to follow scientists and engineers through society.

[CR22] Nihlén Fahlquist J (2006). Responsibility ascriptions and public health problems. Journal of Public Health.

[CR23] Pols, A. (2010). “Transferring responsibility through use plans.” In *Philosophy and engineering: An emerging agenda*, edited by Ibo Poel and David Goldberg, 189–203. Dordrecht: Springer Netherlands. 10.1007/978-90-481-2804-4_16.

[CR24] Robaey Z (2014). Looking for moral responsibility in ownership: A way to deal with hazards of GMOs. Journal of Agricultural and Environmental Ethics.

[CR25] Robaey Z (2016). Gone with the wind: Conceiving of moral responsibility in the case of GMO contamination. Science and Engineering Ethics.

[CR26] Schmidt M (2008). Diffusion of synthetic biology: A challenge to biosafety. Systems and Synthetic Biology.

[CR27] Shelley-Egan C, Bowman M (2015). The challenge of distributing regulatory responsibilities for unknown risks: ‘Nano’-cosmetics and the EU cosmetics regulation as a case study. Journal of Clinical Research & Bioethics.

[CR28] Van de Poel I, Vincent N, Van de Poel I, Van den Hoven J (2011). The relation between forward-looking and backward-looking responsibility. Moral responsibility.

[CR29] Van de Poel I (2011). Nuclear energy as a social experiment. Ethics, Policy & Environment.

[CR30] Van de Poel, I. (2013a) Translating values into design requirements. In Diane P. Michelfelder, Natasha McCarthy & David E. Goldberg (Eds.), *Philosophy and engineering: Reflections on practice, principles and process*. Philosophy of Engineering and Technology 15, (pp. 253–266). Netherlands: Springer http://link.springer.com/chapter/10.1007/978-94-007-7762-0_20.

[CR31] Van de Poel I (2013). Why new technologies should be conceived as social experiments. Ethics, Policy & Environment.

[CR32] Van de Poel I, Fahlquist JN, Doorn N, Zwart S, Royakkers L (2012). The problem of many hands: Climate change as an example. Science and Engineering Ethics.

[CR33] Van de Poel, I., & Kroes, P. (2014) Can technology embody values?” In P. Kroes & P.-P. Verbeek (Eds.), *The moral status of technical artefacts*, Philosophy of Engineering and Technology 17. (pp. 103–124). Netherlands: Springer http://link.springer.com/chapter/10.1007/978-94-007-7914-3_7.

[CR34] Van de Poel I, Royakkers L, Zwart S (2015). Moral responsibility and the problem of many hands.

[CR35] Wetmore JM (2008). Engineering with uncertainty: Monitoring air bag performance. Science and Engineering Ethics.

[CR36] Winner L (1980). Do artifacts have politics?. Daedalus.

